# Loneliness, affectivity and psychological distress in people living alone during the COVID-19 pandemic: a cluster analysis

**DOI:** 10.1186/s41155-026-00392-3

**Published:** 2026-05-08

**Authors:** Kleber Roberto da Silva Gonçalves de Oliveira, Fabio Alexis Rincón Uribe, Edson Júnior Silva da Cruz, Janari da Silva Pedroso

**Affiliations:** 1 Programa de Pós-Graduação em Psicologia, Universidade Federal do Pará, Belém, Pará Brasil; 2 Programa de Pós-Graduação em Psicologia and Programa de Pós-Graduação em Teoria e Pesquisa do Comportamento, Universidade Federal do Pará, Belém, Pará Brasil

**Keywords:** Loneliness, Social isolation, Positive affect, Negative affect, Pandemic, COVID-19

## Abstract

**Introduction:**

During the COVID-19 pandemic, rates of loneliness increased significantly due to the challenging and adverse context arising from this global health emergency.

**Objective:**

To classify adults living alone according to perceived loneliness and affectivity and to examine their association with symptoms of psychological distress during the COVID-19 pandemic.

**Method:**

The design of this study was cross-sectional, descriptive and correlational. The sample consisted of 418 adults living alone, recruited through a non-probabilistic snowball sampling procedure using an online survey distributed via social media and email. To analyze the data, a cluster analysis was applied using the k-means method, the chi-squared test and the Odds Ratio statistic.

**Results:**

Two homogeneous groups were identified: Group 1 (n = 233) characterized by high levels of perceived loneliness, low levels of positive affect and high levels of negative affect; Group 2 (n = 185) showed low levels of perceived loneliness, high levels of positive affect and low levels of negative affect. Cluster 1 was more likely to have symptoms of psychological distress than cluster 2.

**Conclusions:**

To summarize, loneliness among individuals who reside alone can have an impact on the likelihood of experiencing psychological distress and adverse effects.

## Introduction

With the advent of the COVID-19 (Coronavirus disease 2019) pandemic, research has focused on examining how this global emergency event has affected various aspects of physical and mental health. In the early stages of the pandemic, distance restrictions, social isolation, fear of infection, and uncertainty changed people’s lifestyles (Musa et al., 2023). Consequently, social interactions were disrupted, work environments were substituted with remote work, schools and universities were closed, and daily life was conducted entirely at home (Scapaticci et al., 2022). This allowed people to remain in safe environments, subject to preventive measures to inhibit the growth of the disease, such as social distancing measures and quarantines. Although these were effective in the early stages of the outbreak, over the course of the pandemic, they had detrimental and damaging consequences for people in all nations.

In the Brazilian context, the pandemic was not only a public health crisis but also a phenomenon marked by political and institutional tensions, characterized by weaknesses in federal coordination, public disputes over distancing measures, and inconsistent institutional messaging, which contributed to a scenario of uncertainty in the management of public health actions (Barberia & Gómez, 2020; Ortega & Orsini, 2020). This context was associated with the rapid spread of the virus, high mortality rates, and unequal impacts across regions and social groups (Colonia et al., 2023). In such a scenario, it is plausible that individuals living alone experienced more intensely the reduction of face-to-face interactions and the weakening of support networks, contributing to higher levels of loneliness, negative affect, and psychological distress.

In this context, one of the most frequent experiences was loneliness, understood as a subjective experience characterized by the perception of differences between actual and desired social relationships, as well as dissatisfaction with the quantity and quality of these relationships (Cacioppo et al., 2014). A clear distinction must be made between loneliness and social isolation, which refers to the objective absence or paucity of interaction between a person and their social group (Gardiner et al., 2018). It is important to note that, in pre-pandemic times, loneliness was a public health problem and was considered a priority in global public health (World Health Organization [WHO],^ 2023^). However, during the pandemic, loneliness rates increased due to social isolation and quarantine measures (Lederman, 2023). Some longitudinal evidence has shown that, compared to measures collected before this health emergency, there was a significant increase in feelings of loneliness at the beginning and during the pandemic in different groups (Bu et al., 2020; McGinty et al., 2020).

In contrast, some evidence shows that people experience a significant increase in feelings of social connectedness during the pandemic, meaning that social life has not changed significantly (Folk et al., 2020). A study carried out by Luchetti et al. (2020) with 1545 people aged between 18 and 98 compared levels of loneliness in the first, second and third waves of the pandemic, which showed that there were no significant differences. In this sense, it is important to note that even if social interactions were interrupted, the quality of these interactions did not seem to change (van Tilburg et al., 2021). The search for connection has led to the discovery of other forms of communication, with online interaction via a smartphone and phone calls being the main forms of communication (Perez-Brumer et al., 2022).

The characteristics that predicted a greater perception of loneliness during the pandemic were: being young, having a low income, low schooling, being unemployed, living in an urban area and being a student, with living alone being a relevant risk factor that significantly increased this experience (Bu et al., 2020; Li & Wang, 2020). Evidence has shown that people living alone during the pandemic had a worse mental state, with more psychological distress and physical health problems, as well as limited access to relationships and social activities (Kikuchi et al., 2022; Lewis et al., 2023; Noguchi et al., 2023). However, some evidence has shown that some people who live alone have been motivated to seek and establish a social relationship and receive help, which has led them to have greater satisfaction with life and levels of positive emotions (Fingerman et al., 2021; Lee et al., 2024). Therefore, for some people who live alone, living alone was an opportunity to seek out social contact and this allowed feelings of social isolation and loneliness to diminish.

In recent years, there has been an 80% increase in the number of people choosing to live alone, from 153 million in 1996 to 227 million in 2011 (Spence et al., 2019). According to some evidence, people’s decision to live alone can be motivated by a variety of reasons, including a voluntary and motivated desire to achieve personal results and goals (Li & Kanazawa, 2016; Mauss et al., 2012), as well as major events such as widowhood, old age or financial problems (OʼSúilleabháin et al., 2019). Living alone can be linked to both positive and negative outcomes. Exposure to stressful events such as the COVID-19 pandemic has affected people’s feelings of loneliness and solitude in various ways, which may have helped to mitigate the effects of these adverse events.

Although the data were collected during the initial phase of the COVID-19 pandemic (March–May 2020), this period represents a critical context marked by high uncertainty and abrupt social changes, allowing for the examination of psychological responses under conditions of heightened vulnerability. Insights from this period may inform future public health responses, particularly regarding mental health monitoring and remote support strategies for individuals living alone. In this context, the aim of this study was to classify adults living alone into homogeneous groups based on their levels of perceived loneliness, positive affect, and negative affect; and to analyse the association of these groups with symptoms of psychological distress during the COVID-19 pandemic.

## Methods

### Participants

This study included 418 Brazilian adults who reported living alone, drawn from a larger international survey. Non-probabilistic sampling was adopted, and the snowball technique was used to recruit participants. This selection process was facilitated by the researchers and key participants, who invited potential participants online (invitation on social networks and by e-mail). Participants were included if they were over 18, lived alone, and agreed to participate.

## Measures

### Positive affect and negative affect

Affects were assessed in terms of the level of intensity experienced in Positive Affect (PA) and Negative Affect (NA), using the short version of the PANAS (Positive Affect and Negative Affect Scale) (Watson et al., 1988). The PANAS is a self-report scale with two subscales: a measure of PA and a measure of NA, each with 10 items. In this study, an abbreviated version was used which includes 5 items for PA (Interested, Enthusiastic, Inspired, Active and Determined) and 5 items for NA (Frightened, Scared, Anguished, Irritated and Nervous). Each item was evaluated using a five-point Likert scale, ranging from 1 “Not at all” to 5 “Extremely”. The total score for each subscale is obtained from the sum of the items that make up each construct, with higher scores being interpreted as a greater presence of affect (Roemer & Medvedev, 2023). The abbreviated PANAS was administered in Portuguese, including five items for positive affect and five items for negative affect, as described above. The PA and NA subscales showed acceptable internal consistency in this sample (PA: α = 0.865, 95% CI: 0.844–0.884; NA: α = 0.900, 95% CI: 0.884–0.914).

### Perception of loneliness

The perception of loneliness was measured using the item: “Most of the time I feel lonely”. The response options were based on a 5-point Likert scale, where 1 = “Totally false”, 2 = “False, most of the time”, 3 = “Neutral”, 4 = “True, most of the time” and 5 = “Totally true”. Higher scores are considered a high perception of loneliness. Although single-item instruments do not allow for an assessment of internal consistency, they have been widely used in large-scale population studies and have demonstrated acceptable validity in measuring subjective experiences such as loneliness (Baerg MacDonald & Schermer, 2025; Reinwarth et al., 2023).

### Symptoms of psychological distress

A questionnaire was created to identify the presence or absence of 10 symptoms of psychological discomfort: insomnia, tachycardia, nausea, headache, restlessness, change in appetite, muscle tension, tiredness, wanting to cry and excessive fear. This list included a single question: “In the last week I have presented” and the response options were made in a dichotomous Likert format, “yes or no” indicating the presence or absence of the symptom, respectively. The selection of symptoms was informed by prior literature on psychological and somatic responses during the COVID-19 pandemic (Holingue et al., 2020). This checklist was used as an indicator of self-reported psychological symptoms and should not be interpreted as a diagnostic or psychometrically validated measure of psychological distress.

### Procedure

This study is part of an international study (information removed for review). A virtual questionnaire was applied using the SurveyMonkey online platform, and data was collected between March and May 2020, during the first wave of the COVID-19 pandemic. The invitation and sample selection process were carried out through a virtual environment (social networks and e-mail addresses). Participants had between 10 and 15 min to answer the questionnaire and the answers were stored in a general database. The overall sample consisted of 5,499 participants. For the purposes of this study, only respondents who reported living alone were selected, resulting in a subsample of 418 adults. Finally, the data was analyzed using R Software and R-Studio (version 4.2.3).

### Statistical analysis

In this study, a cluster analysis was carried out and k-means was used as the method for identifying and forming the groups. Prior to cluster analysis, all continuous variables (perceived loneliness, positive affect, and negative affect) were standardized (*z*-scores) to identify potential outliers, defined as *z*-scores greater than 3 or less than − 3 (Salgado et al., 2016). Subsequently, to assess the validity of the clusters, the silhouette index was calculated. This parameter can vary between − 1 and 1 (where values > 0.30 are considered adequate) and uses the closest distance to the cluster and the average dissimilarity of the observations as two grouping criteria (Lengyel & Botta-Dukát, 2019). To check whether the groups created showed significant differences in relation to the measures used for grouping, the Mann-Whitney U-test was applied. The chi-squared test was used to determine the association between sociodemographic variables and the distribution of clusters (cluster 1 and cluster 2). The chi-square test (X^2^) is a non-parametric technique (without distribution) and is used to measure the association between categorical variables in the same population (McHugh, 2013). The significance level was set at *p* < .05.

To assess the probability of symptoms for the clusters, the odds ratio (OR) was calculated. The OR is a statistic used to show the strength of the association between the exposure event and the presence or absence of an outcome (e.g. a disease) (Park & Han, 2022). The exposure event was the distribution of participants in the clusters (considering high or low levels of perceived loneliness, positive and negative affect), while the outcome or event of interest was self-reported psychological symptoms (determined as present or absent). It is important to note that analysis of associations between clusters and symptoms were not adjusted for potential sociodemographic confounders. Therefore, the results should be interpreted as unadjusted associations. To interpret the strength of the OR, the magnitude of the effect (ME) was considered, which represents the strength of the association between the event and the specific risk (groups). Thus, the following levels of interpretation were considered: insignificant (< 1.68), small (between 1.68 and 3.47), moderate (between 3.47 and 6.71) and large (> 6.71) (Chen et al., 2010). The level of statistical significance was determined using the confidence intervals of the ORs that did not include the 1st OR. All statistical analyses were conducted in R and R Studio.

## Results

The sample consisted of 418 Brazilian adults who were surveyed online during the first peak of the COVID-19 pandemic. The gender distribution was made up of 68% women, 32% men and an overall mean age of 41.78 (SD = 14.35), with ages ranging from 18 to 60 years. 61% of the participants were employed and 40% reported having an income of between 300 and 900 dollars. During this period of the pandemic, 62% reported being in quarantine and 65% reported being in preventive social isolation for more than 15 days.

### Cluster analysis

Initially, no outliers were identified based on the standardized score criterion (z-scores greater than 3 or less than − 3). In the cluster analysis, clusters were identified using the following input variables: perception of loneliness, positive affect and negative affect. The classification obtained using the K-means indicated that the participants were grouped into two clusters, which explained 84.71% of the variability in the observations. According to the Silhouette index, the average cohesion and separation was 0.36, which indicates that the observations were adequately grouped. Thus, the validation of the clusters indicated that the input measures are statistically different and discriminating for the scores of perceived loneliness (W = 38507.500; *p* < .001), positive affect (W = 9304.000 *p* < .001) and negative affect (W = 40521.000; *p* < .001). Cluster 1 had high levels of perceived loneliness (M = 3.91; SD = 0.96), low levels of positive affect (M = 12.53; SD = 3.89) and high levels of negative affect (M = 16.42; SD = 3.93). Cluster 2 had low levels of perceived loneliness (M = 2.02; SD = 0.98), high levels of positive affect (M = 16.74; SD = 3.76) and low levels of negative affect (M = 8.94; SD = 2.95).

Among the clusters identified, cluster 1 (*n* = 233) was characterised by being female (70%), employed (66%), with an income of more than USD (United States Dollar) 1200 (53%), with between 13 and 18 years of schooling (55%) and with a house size of up to 50 m2 (44%). Meanwhile, cluster 2 (*n* = 185) was characterised by being female (67%), employed (55%), with an income of more than US 1200 (69%), with between 13 and 18 years of schooling (44%) and with a house size of between 50 m^2^ and 80 m^2^ (34%). In addition, cluster distribution was significantly related to occupation (X^2^ = 47.728, *p* = < 0.001), income (X^2^ = 12.349, *p* = < 0.001) and house size (X^2^ = 18.580, *p* = < 0.001). Details of the sociodemographic data are available in Table 1.

**Table 1:** Socio-demographic characteristics of the clusters

	Cluster 1 *n* = 233 (56%)	Cluster 2 *n* = 185 (44%)		
	f (%)	f (%)	X^2^	*p*-value
Sex				
Male	71 (30%)	62 (33%)		
Female	162 (70%)	123 (67%)	0.449	0.799
Occupation				
Self-employed	21 (9%)	27 (15%)		
Retired	7 (3%)	35 (19%)		
Unemployed	14 (6%)	5 (3%)		
Employed	154 (66%)	102 (55%)		
Entrepreneur	3 (1%)	8 (4%)		
Student	34 (15%)	8 (4%)	47.728	< 0.001
Income				
≤ 300	53 (23%)	34 (18%)		
> 300 ≤ 600	50 (21%)	36 (20%)		
> 600 ≤ 900	47 (20%)	33 (18%)		
> 900 ≤ 1200	30 (13%)	13 (7%)		
> 1200	53 (23%)	69 (37%)	12.349	< 0.001
Number of years of schooling				
0	1 (0.4%)	3 (2%)		
1 to 12 years old	13 (5.6%)	10 (6%)		
13 to 18 years old	127 (55%)	82 (44%)		
19 to 22 years old	61 (26%)	58 (31%)		
23 and over	31 (13%)	31 (17%)	5.735	0.220
House size				
Up to 50 m2	102 (44%)	53 (29%)		
Between 50 and 80 m2	85 (37%)	63 (34%)		
Between 80 and 120 m2	31 (13%)	42 (23%)		
More than 120 m2	15 (6%)	27 (14%)	18.580	< 0.001

*m*^2^  square metres,*f*  frequencies, *%* percentages,*X*^2^ chi-squared coefficient

As for the prevalence of symptoms of psychological discomfort, Table 2 shows that the symptoms with the highest prevalence (≥ 50%) in the adults grouped in cluster 1 were restlessness (76%), insomnia (62%), wanting to cry (61%), tiredness (52%) and headache (50%), while muscle tension (48%), change in appetite (48%), excessive fear (37%), tachycardia (23%) and nausea (13%) were the symptoms with the lowest prevalence. With regard to the participants in cluster 2, the prevalence of symptoms was no higher than 50 per cent and those with the highest proportion were restlessness (28 per cent), insomnia (27 per cent), tiredness (25 per cent), muscle tension (23 per cent) and headache (20 per cent), while the desire to cry (17 per cent), change in appetite (16 per cent), nausea (3 per cent), excessive fear (3 per cent) and tachycardia (3 per cent) manifested themselves to a lesser extent (see Table 2).

**Table 2:** Prevalence of symptoms of psychological distress

Symptoms	Cluster 1 (*n* = 233)		Cluster 2 (*n* = 185)					
	Yes *f (%)*	No *f (%)*	Yes *f (%)*	No *f (%)*	OR*	*95% CI*		*ME*
						*Lower*	*Upper*	
Fatigue	122 (52%)	111 (43%)	46 (25%)	139 (75%)	3.321	2.180	5.060	Small
Headache	116 (50%)	117 (50%)	38 (20%)	147 (80%)	3.835	2.471	5.952	Moderate
Restlessness	178 (76%)	55 (24%)	52 (28%)	133 (72%)	8.278	5.328	12.861	Large
Insomnia	144 (62%)	89 (38%)	50 (27%)	135 (73%)	4.369	2.875	6.639	Moderate
Excessive fear	88 (37%)	145 (63%)	6 (3%)	179 (97%)	18.106	7.696	42.594	Large
Change in appetite	113 (48%)	120 (52%)	29 (16%)	156 (84%)	5.066	3.159	8.124	Moderate
Nausea	30 (13%)	203 (87%)	5 (3%)	180 (97%)	5.320	2.021	14.003	Moderate
Tachycardia	54 (23%)	179 (77%)	6 (3%)	179 (97%)	9.000	3.776	21.450	Large
Muscle tension	113 (48%)	120 (52%)	42 (23%)	143 (77%)	3.206	2.087	4.925	Small
Wish to cry	143 (61%)	90 (39%)	31 (17%)	154 (83%)	7.893	4.946	12.696	Large

*CI*  Confidence Interval, * ME*  Measure of Effect, *OR*  Odds Ratio

Considering these proportions, the results shown in Table 2 indicate that people with low levels of positive affect, high levels of perceived loneliness and negative affect are more likely to reports psychological symptoms. Thus, the prevalence ratio indicates that the symptoms most likely to occur in this population group are excessive fear (OR = 18.106: 95% CI 7.696–42.594), tachycardia (OR = 9.000 95% CI 3.776–21. 450), restlessness (OR = 8.278; 95% CI 5.328–12.861), desire to cry (OR = 7.893; 95% CI 4.946–12.696), nausea (OR = 5.320; 95% CI 2.021–14.003) and change in appetite (OR = 5.066; 95% CI 3.159–8.124) (See Table 2). All the results were statistically significant.

## Discussion

The results of this study showed that, in a sample of adults who declared they lived alone, there were two statistically different groups, the first with high levels of perceived loneliness (cluster 1) and the second with low levels of loneliness (cluster 2). According to some evidence, the two groups identified are similar to the typologies found in latent class analysis studies, which showed homogeneous groups of participants according to levels of loneliness and isolation, such as: Not Lonely-Alone, Not Lonely-Alone, Lonely-Connected and Lonely-Alone-Other (Hsu, 2020; Smith & Victor, 2019). This may suggest that even though people live alone, it doesn’t mean they experience loneliness and can be connected and actively interact with others. This suggests that loneliness and isolation are different experiences, and that loneliness can be influenced by a range of personal, social and contextual factors.

Although the classification in this study is similar to that found in the literature, it is important to emphasise that the social isolation imposed on the participants was due to a global health emergency. Thus, the two groups found could be characterised as: (1) preventively isolated, lonely and with a high perception of loneliness and (2) preventively isolated, lonely and with a low perception of loneliness. According to some studies, the description of group 1 may indicate that living alone can lead people to feel less connected to other people, more lonely and less likely to socialise, which increases the risk of experiencing loneliness (OʼSúilleabháin et al., 2019; Shaw et al., 2021). In contrast, in group 2, there was evidence that living alone encouraged social contact (through phone calls or video calls, for example) and consequently favoured a decrease in perceived loneliness compared to those who lived with other people and experienced significant levels of loneliness (Castillo et al., 2022; Fingerman et al., 2021).

In the groups identified, group 1, characterised by the highest levels of loneliness, showed a greater intensity and frequency of negative affections compared to positive affections. The results corroborate the idea that the experience of loneliness generates a series of affective biases that generate a series of deregulated affective responses that can include feelings of sadness, depression and frustration (Hawkley & Cacioppo, 2010). Furthermore, this characterization agrees conceptually with loneliness, as the focus of this definition is negative affect and this experience is described as a feeling of distress that is linked to the perception that social needs are not being met (Cacioppo & Hawkley, 2009). Based on this definition, the absence of positive affect could also be a trait that differentiates those who experience this feeling of loneliness.

According to ample empirical evidence, these results confirm that feelings of loneliness are positively related to negative feelings and negatively related to positive feelings (Gniewosz, 2023; Newman & Sachs, 2020; Steptoe, 2023). The relationship between affectivity and loneliness can be explained by the fact that experiencing negative affections can decrease interest in social interactions and cause more emotional distress, which in turn increases the likelihood of experiencing loneliness (Luo & Shao, 2023; Wolters et al., 2023). In this way, it seems that positive affect helps social interaction and bonds with other people, while negative affect harms the quality of social interactions, decreases the frequency of participation in social events and limits social contact.

As has already been discussed, loneliness is associated with a higher frequency of negative affections compared to positive affections, but it seems that context plays a relevant role in this relationship. During the pandemic, the need for social isolation was imposed as a preventative measure, where people should avoid interaction and direct physical contact. This measure was able to slow down the increase in cases of infection, having negative effects on various areas of human life, especially health, well-being and social relationships (Lloyd et al., 2023; Moura et al., 2022). The first empirical evidence on the effects of the Pandemic revealed that social isolation significantly increased people’s levels of loneliness, which in turn became a risk factor for a range of physical, emotional and psychological problems and symptoms (Lederman, 2023). According to an experimental study conducted by Ditcheva et al. (2018), a group of individuals subjected to social threat induction and social disconnection showed lower positive affect reactivity and higher negative affect reactivity. These results corroborate the idea that context has a relevant influence on the relationship between the experience of loneliness and affectivity.

During the pandemic, several studies have shown high self-reposted psychological symptoms and psychological discomfort in populations from different countries (Marzo et al., 2021). Psychological distress is generally triggered by exposure to stressful or challenging events (Holingue et al., 2020), when these are presented alongside other negative experiences (e.g. loneliness or psychological disorders) they increase the risk of symptoms manifesting more frequently, for longer and more intensely (Kim et al., 2021). Some studies have shown that those who suffer from more severe psychological problems, compared to those without such problems, have higher rates of psychological distress or discomfort, with insomnia, depression, headache, tidal wave, muscle pain and suicidal ideas being the most frequent symptoms (Kippe et al., 2023; Oppenauer et al., 2021). Considering this, the results of this study showed significant self-reported psychological symptoms in the homogeneously identified groups.

Participants in group 1 were more likely to show symptoms of psychological discomfort, such as fear, tachycardia, the urge to cry and restlessness, compared to participants in group 2. Some neurophysiological studies show that individuals who suffer from loneliness show greater physiological changes, including greater total basal peripheral resistance, changes in the autonomic nervous system and increased cardiac expenditure. This leads to a greater manifestation of psychopathological symptoms and less favourable evaluations in social interactions (Cacioppo et al., 2000; Hawkley et al., 2003). In addition, it has been shown that loneliness is linked to altered cortisol levels, with lower levels during the day and afternoon, and higher levels at night, which results in a greater reaction to external stimuli (Vitale & Smith, 2022). In this way, it can be inferred that loneliness can affect people’s physical health and consequently cause symptoms that can be linked to stress, anxiety or depression.

So, in individuals who live alone is physiological reactivity more intense compared to those who live alone? According to a study carried out by Cacioppo et al. (2000) on a sample of university adults, the results showed that both lonely and non-lonely students had the same chance of doing restorative activities (for example, exercising, sleeping or interacting with other people), but these activities did not decrease the physiological reactivity associated with stress. In this way, it can be surmised that lonely people, unlike non-lonely people, experience greater intensity of physiological symptoms related to stress or anxiety, which increases feelings of anguish and nervousness resulting from stress.

The social isolation caused by the pandemic prevented social interaction between individuals, which had an impact on people’s mental health and well-being. Several studies corroborate the results of this study, demonstrating a significant and positive relationship between feelings of loneliness and symptoms associated with distress and psychological discomfort during the period of confinement imposed by the Pandemic (Keller et al., 2023; Latikka et al., 2022). In addition, a study carried out by Benke et al. (2020) found that, during the social isolation of the COVID-19 Pandemic, people who reported greater distress or psychological discomfort also reported a lower frequency of weekly contact with other people, which was associated with levels of loneliness and symptoms of anxiety and depression. Thus, this may indicate that the context social isolation was associated with psychological discomfort and loneliness, rather than establishing a causal relationship between these variables. This, in turn, may have been associated with people’s feelings of loneliness, which increased levels of loneliness, especially in lonelier people.

Furthermore, the results of this study also revealed that lonely individuals exposed to this challenging environment presented lower levels of loneliness. Participants in group 2 were statistically homogeneous in relation to group 1, with lower rates of symptoms related to psychological distress, which reduces the chance of presenting these symptoms. In the literature, it is widely demonstrated that social connection and support have beneficial effects on several aspects related to physical and mental health and well-being, while loneliness has negative effects associated with the emergence of diseases, mental disorders and adverse social problems (Martino et al., 2015; Shah & Househ, 2023). However, previous evidence, in line with the results of this research, indicates that loneliness may also be associated with a lower chance of suffering from physical or mental illnesses, either through the ability to cope with the negative effects of loneliness or through the exploration of emotional benefits (e.g., living meaningful individual activities, times of reflection) (Lin et al., 2020; Rodríguez et al., 2020).

The main merit of this study was the use of a robust analysis to group in a statistically homogeneous manner a set of self-reported responses on the experience of loneliness, the experience of affects, and their relationship with the manifestation of symptoms of psychological distress. Thus, cluster analysis is considered a highlight, since this analytical technique encompasses several rigorous and widely documented statistical methods, which can contribute to the improvement and complementarity of results derived from other methods (Frades & Matthiesen, 2010). The analysis allowed the identification of two statistically homogeneous groups, consistent with previous literature. In addition, the data were collected in the first period of the pandemic (between March and April 2020), which allowed an approximate understanding of how people feel in social isolation, their feelings and symptoms immediately after the imposition of social isolation.

However, this result should also be considered due to its limitations. One important limitation relates to the context in which the data were collected. This study was conducted during the COVID-19 pandemic, a period characterized by social restrictions and lockdowns. Therefore, the levels of loneliness and the magnitude of the effects in this study may have been influenced by factors specific to the pandemic, such as lockdown measures, reduced in-person interactions, and fear of infection. Thus, the results may have limited external validity and should be interpreted with caution when generalized to non-pandemic contexts. Second, the assessments were based on a sample that is not representative of the population, which prevents the generalization of the results. Furthermore, there was no probabilistic sampling of participants, which could have favored the composition of a sample that is significant in relation to the population. Third, the symptom checklist used in this study was not a psychometrically validated measure, which may affect the precision and interpretability of the results. Future studies should employ standardized and validated instruments to assess psychological distress and related constructs.

Finally, another limitation of this study is the absence of relevant contextual and psychosocial variables. No information was collected on the participants’ place of residence (e.g., urban versus rural settings or large metropolitan areas), which could be important, given that evidence suggests the impact of the COVID-19 pandemic on mental health was more pronounced in densely populated urban settings. Furthermore, living alone was treated as a homogeneous experience, although it may reflect circumstances such as voluntary choice versus obligation, availability of social support, or working conditions. Future studies should incorporate a broader range of contextual and psychosocial variables such as place of residence, perceived social support, frequency and quality of social interactions, and prior physical and mental health conditions. These factors may help explain individual differences in the experience of loneliness and its association with psychological outcomes.

## Conclusions

The results of this study showed that there are homogeneous groups, which reflect how a sample of individuals preventively isolated during the pandemic experience loneliness, depending on the intensity of their negative and positive affect. Two groups were identified: the first, characterized by high perceived loneliness in interaction with high levels of negative affect and low levels of positive affect; the second, characterized by low perceived loneliness in interaction with high levels of positive affect and low levels of negative affect. Thus, when analyzing this grouping, it was found that group 1 had a greater probability of reporting symptoms of psychological symptoms in relation to group 2, which had a statistically lower probability. In addition, it was observed that some sociodemographic factors are related to loneliness, such as income, occupation and size of residence, while sex and education did not have a significant relationship.

## Data Availability

The datasets generated and/or analysed during the current study are available in the OSF repository, https://osf.io/m4r2y/?view_only=29defba4cc6948b2bbe92d56b0dd97f3.

## Abbreviations

COVID-19: Coronavirus disease 2019
ME: Magnitude of the effect
NA: Negative Affect
OR: Odds Ratio
PA: Positive Affect
PANAS: Positive Affect and Negative Affect Scale
USD: United States Dollar
WHO: World Health Organization
